# The patient safety culture as perceived by staff at two different emergency departments before and after introducing a flow-oriented working model with team triage and lean principles: a repeated cross-sectional study

**DOI:** 10.1186/1472-6963-14-296

**Published:** 2014-07-09

**Authors:** Lena Burström, Anna Letterstål, Marie-Louise Engström, Anders Berglund, Mats Enlund

**Affiliations:** 1 Centre for Clinical Research, Uppsala University, Västmanlands County Hospital, Västerås, Sweden; 2 Department of Emergency Medicine, Karolinska University Hospital, Stockholm, Sweden; 3 Department of Medicine, Karolinska Institutet, Solna, Sweden; 4 Department of Surgical Sciences, Uppsala University, Uppsala University Hospital, Uppsala, Sweden

**Keywords:** Patient safety, Patient safety culture, Patient safety climate, Quality improvement, Team-work

## Abstract

**Background:**

Patient safety is of the utmost importance in health care. The patient safety culture in an institution has great impact on patient safety. To enhance patient safety and to design strategies to reduce medical injuries, there is a current focus on measuring the patient safety culture. The aim of the present study was to describe the patient safety culture in an ED at two different hospitals before and after a Quality improvement (QI) project that was aimed to enhance patient safety.

**Methods:**

A repeated cross-sectional design, using the Hospital Survey On Patient Safety Culture questionnaire before and after a quality improvement project in two emergency departments at a county hospital and a university hospital. The questionnaire was developed to obtain a better understanding of the patient safety culture of an entire hospital or of specific departments. The Swedish version has 51 questions and 15 dimensions.

**Results:**

At the county hospital, a difference between baseline and follow-up was observed in three dimensions. For two of these dimensions, *Team-work within hospital and Communication openness*, a higher score was measured at the follow-up. At the university hospital, a higher score was measured at follow-up for the two dimensions *Team-work across hospital units* and *Team-work within hospital.*

**Conclusion:**

The result showed changes in the self-estimated patient safety culture, mainly regarding team-work and communication openness. Most of the improvements at follow-up were seen by physicians, and mainly at the county hospital.

## Background

### Patient safety and medical injuries

Patient safety can be described as the avoidance, prevention and amelioration of adverse outcomes or injuries that stem from the process of healthcare [1]. Patient safety is of the utmost importance for health care, and the World Health Organization has stated that patient safety is a fundamental principle of health care. In Sweden, this is regulated by law [2].

A survey by the Swedish National Board of Health and Welfare showed that almost 9% of Swedish patients in somatic in-patient care experienced a preventable adverse event [3]. Failures in communication and team-work are frequent contributors to medical injuries in health care [4]. Communication failures are the leading causes of inadvertent patient harm. According to the Joint Commission on Accreditation of Healthcare Organizations in the United States of America (USA), 70% of all negative events are caused by communication failures [5]. The lack of standardized procedures and standardized ways to communicate in health care increases the importance of the creation of a common mental model for communication [5]. Increased leadership standards, team-work and multidisciplinary collaboration in patient care are associated with lower mortality rates and reductions in hospital stay [6-8].

### Patient safety in the emergency department (ED)

Since the majority of the patients come to an ED with symptoms and without a clear-cut diagnosis, it is imperative that health care professionals are highly skilled and well trained to perform high quality care based on basic principles. Common problems and deficiencies in the ED that may lead to patient safety risks or damage are related more to overcrowding, communication failure in connection with transfers and a lack of team-work, than to medical mishaps or lack of knowledge [9]. Handover and transition of patients have become focuses of efforts towards reducing errors. Up to 50% of errors in communication occur during hand-off [9,10]. Thus, patient hand-offs at shift changes in an ED are an important safety process and a critical moment [11]. The safety aspects of the transfer of patients from an ED to an admitting physician are insufficiently studied. However, associations with adverse events have been observed when patients are transferred from the ED to an internal medicine ward [9]. Specifically vulnerable areas include patient flow, work-load, communication, information technology, assignments of responsibility and environment. In addition, the working environment, which demands constant multitasking, is a challenge [12]. System-based interventions may prevent many of these adverse events and consequently improve patient safety [9,13].

### Patient safety culture

The patient safety culture in an institution has a great impact on patient safety [14]. To enhance patient safety and design strategies to reduce medical injuries, there is a current focus on measuring the patient safety culture [14,15]. The patient safety culture is a component of “organisational culture” and reflects the shared beliefs, attitudes, values, norms and behavioural characteristics of individuals [15,16]. Moreover, it influences staff member attitudes and behaviours in relation to their organisations’ on-going patient safety performance [17-19]. The patient safety climate is another component of the conception of “organisational culture” and the terms “culture” and “climate” are now used interchangeably in the literature.

It is difficult to define measurable components of the patient safety culture [20], and because of this, a number of patient safety climate questionnaires have been developed [21,22]. One of these questionnaires, the Hospital Survey on Patient Safety Culture (HSOPSC) [21,23,24], is recommended for use and has been translated into Swedish by a national group [25] .

The components that are measured in this questionnaire could be described as the employee’s perceptions and attitudes about the surface features of the patient safety culture [21,26]. Questionnaires can also be used to examine the effectiveness of strategies designed to improve the patient safety culture and patient safety. However, the evidence supporting the effectiveness of these strategies within hospitals is limited [15].

The aim of the present study was to describe the patient safety culture in an ED at two different hospitals before and after a Quality improvement (QI) project that was aimed to enhance patient safety.

## Methods

### Study design and instrument

A repeated cross-sectional design, using the HSOPSC questionnaire before and after a Quality improvement project that aimed to enhance patient safety in the ED at two hospitals, a county hospital and a university hospital. The hospitals are located in two different cities in central Sweden.

### County hospital

The county hospital is a trauma level II centre located in a minor city, covering a source population of 251,000. The ED at this hospital has an average of 53,000 attending patients annually. It serves adults and children in four specialties: medical, surgical, orthopaedic and gynaecology. In 2009, at the first questionnaire survey, the ED worked with traditional single nurse triage. After triage, a junior physician examined the patients. At the commencement of the study, the ED used a locally-modified version of the Manchester Triage Scale [27,28]. The hospital has a fast-track percutaneous coronary intervention (PCI) line for patients with myocardial infarction and another fast-track line for patients with stroke. Both medical and non-medical students are in training in the ED.

### University hospital

The university hospital is a trauma level I centre in the capital city of Sweden. The ED serves a population of 150,000 with medical, surgical and orthopaedic care, and the hospital serves a population of 2 million with specialized care, such as oncology and ear, nose and throat care. The ED at this hospital has an average of 75,000 attending patients annually. In 2008, at the first questionnaire survey, the ED used the Adaptive Process Triage (ADAPT) scale [27,28]. With ADAPT, a patient was assessed using a two-step triage model. In step one, a single nurse assessed all patients, and thereafter they were either referred to a team triage, consisting of a nurse and an emergency physician, or to a senior specialist physician within the ED. The hospital has a fast-track PCI for patients with myocardial infarction and another fast-track line for patients with stroke. The department conducts continuing education of physicians and nurses aiming to become specialists in emergency medicine and emergency nursing, respectively. Swedish and non-Swedish medical and non-medical students are in training in this ED.

### Project aiming for quality improvement at the county hospital

**Figure 1:**
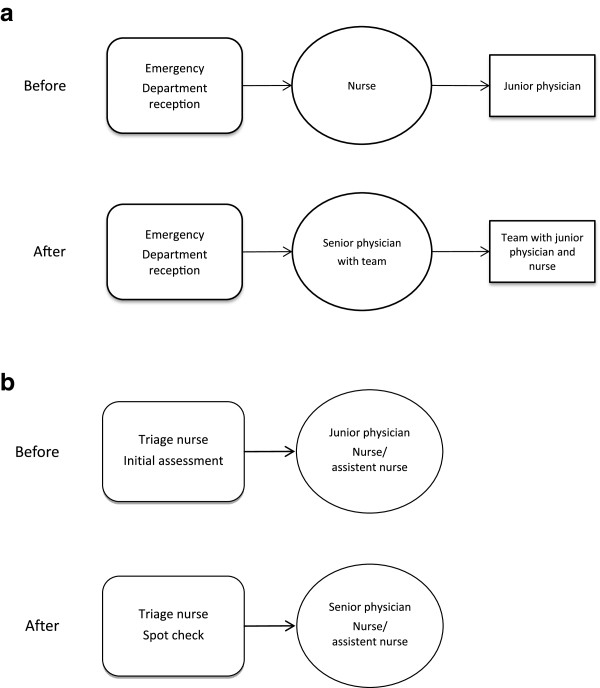
**The principal organisation of the triage models studied. a)** The principal organisation at the ED in the county hospital before and after quality improvement project. **b)** The principal organisation at the ED in the university hospital before and after quality improvement project.

**Figure 2:**
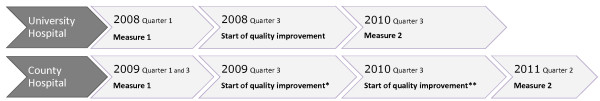
**Study timeline.** *start of quality improvement in the section of medicine. ** start of quality improvement in the section of orthopaedics.

In 2009 (quarter 3), physician triage with a supporting team, based on a flow process and lean principles [29], was introduced to the internal medicine section of the ED in a limited manner. The purpose was to shorten the time to first contact with a physician and thereby lead to a shorter stay and enhanced patient safety in the ED. The model was extended in spring 2010 to be in use from 8:00 to 16:00, and was further modified in May 2011 to be in use from 9:00 to 20:00). In this model, the patients were first triaged by a nurse and, if assessed as internal medicine patients they were transferred directly to a physician, a specialist in internal medicine at the ED.Physician triage was introduced to the orthopaedic section of the ED in 2010 (quarter 3), to be in use from 10:00 to 16:00 (Figure 1a), and to the surgical section in 2012 (quarter 3) for hours 10:00 to 16:00. The ED and the Department of Internal Medicine initiated the project, and the hospital management supported the process (Figure 2).

### Project aiming for quality improvement at the university hospital

A major organisational change was implemented in the ED of the university hospital in 2008, (quarter 3) with the main purpose of enhancing patient safety by improving the patient flow in the ED, hence shortening the total visit time and the time to be seen by a physician. This quality improvement project was based on lean principles [29] and involved all specialities in the ED. The model consisted of an initial assessment (“spot-check”) of the patient by the triage nurse before the patient was referred to a team consisting of a specialist, a nurse and an assistant nurse. If the appropriate team was not available, the triage nurse performed an assessment to determine the acceptable waiting time for the patient to be seen by a physician (Figure 1b). The hospital management initiated the project and supported the implementation process with external facilitators. Improvements were made during the implementation process based on the principles of continuing improvement, using the PDSA cycle (plan, do, study, and act), meaning that adjustments and evaluations are both an on-going process. The roles of the nurses and physicians in the ED within the project were mainly to contribute as members of different local groups (Figure 2).

### Hospital survey using the patient safety culture questionnaire (HSOPSC)

The HSOPSC was developed for the Agency for Healthcare Research and Quality, to obtain a better understanding of the patient safety culture of an entire hospital or of specific departments. HSOPSC has been used primarily for intra- and inter-institutional comparisons [22]. HSOPSC is based on a set of pilot studies carried out in 21 different hospitals across the USA, involving 1,461 hospital staff. The original questionnaire consists of 42 items, which are grouped in 12 dimensions. HSOPSC is a validated and widely used instrument [30]. Blegen et al. have concluded that the subscales measuring of the safety culture dimensions seem to be moderately reliable and valid at both the individual respondent level and at the unit level, for which the questionnaire was designed [26]. However, they showed that the tool was sensitive to differences between hospital units, to differences over time and across disciplines. This tool can therefore be used to describe differences in each dimension of the safety culture across time, discipline, unit or institution.

The questionnaire used in this study has been translated into Swedish and validated by the Swedish National Board of Health and Welfare [25], and it is a modified version of the HSOPSC. Two dimensions have been added to the Swedish version, *Information and support to patients at adverse events* and *Information and support to staff at adverse events*. The questionnaire consists of 51 items, which are aggregated into 15 dimensions to measure respondents’ attitudes about various aspects of patient safety (Table 1). Each dimension includes one to four items with a 5-point Likert scale. Percentages were calculated on the number of responses to specific questions or dimensions. Responses with scores 1 and 2 were considered negative regarding patient safety, 3 was neutral, and 4 and 5 were positive. Some questions were negatively worded, so the answers were reversed prior to recording into positive or negative. The dimensional scores were expressed as percentages of answers within each dimension that indicated a positive response towards patient safety [31]. Statistically significant improvement in outcomes between baseline and follow-up is described as a “higher score” or a “positive score”. In the Swedish manual, an index of < 50 is considered low and should lead to action, 51–69 suggests potential for improvement, and ≥ 70 indicates that the unit is functioning well.

**Table 1:** The content of dimensions in the hospital survey on patient safety culture questionnaire

1. Non-punitive response to error	9. Overall perception of safety
*3 items*	*4 items*
2. Staffing	10. Safety culture dimension at Unit level**
*4 items*	*4 items*
3. Frequency of event reporting	11. Organizational Learning-Continues Improvement.
*3 items*	*3 items*
4. Hospital Management Support for patient safety	12. Teamwork within hospital
*3 items*	*4 items*
5. Teamwork Across Hospital Units	13. Communication openness
*4 items*	*3 items*
6. Hospital Handoff and Transition	14. Feedback and Communication about error
*4 items*	*3 items*
7. Information and support to patients at adverse events*	15. Patient safety grade
*4 items*	*1 item*
8. Information and support to staff at adverse events*	
*2 items*	

*Swedish version.

**Not answered by physicians.

### Data collection and statistical methods

The HSOPSC questionnaire was administered to the staff before and after the quality improvement project, including all registered nurses, assistant nurses, those physicians employed at university hospital ED, and those physicians who frequently work in the ED at the county hospital. Registered nurses and assistant nurses were analysed as one group at each hospital.

The questionnaire was first distributed in 2009 at the county hospital and in 2008 at the university hospital. The second questionnaire was distributed two years after the baseline measurement, i.e., in 2011 and 2010, respectively.

The questionnaire and a cover letter, explaining the purpose of the study, were given to the staff at information meetings. The target group at the county hospital measurement at baseline was 108 registered nurses/assistant nurses and 129 physicians, and at the follow-up there were 114 registered nurses/assistant nurses and 149 physicians. The response rate in the two rounds of questionnaires was 86% and 71%, respectively. The target group at the university hospital measurement at baseline was 125 registered nurses/assistant nurses and 55 physicians and at the follow-up, 125 registered nurses/assistant nurses and 54 physicians. The response rates were 61% and 70%, respectively.

The internal loss of responses was less than 5% for all questions. Physicians at the county hospital did not respond to the questions that built up the dimension number 10, *Safety culture dimension at unit level*, as those questions concern how the employees obtain support from the local management, and the physicians are not employed at the ED. Thus, dimension number 10 was not included in the comparisons involving physicians, and therefore it is 14 dimensions instead of 15 in comparisons in which physicians are included.

### Statistical methods

The proportions of a positive score at baseline and follow-up, by occupation or type of hospital, were analysed using the binominal test. All tests were two-tailed, and *p*-values less than 0.05 were considered statistically significant. The statistical analyses were performed using SAS Statistics software (SAS Institute Inc., Cary, NC, USA).

### Ethics

Verbal and written information regarding the aim and procedure was given to all staff who were informed that they were free to withdraw from the study at any time and without declaring any reason to do so. The written information consent was obtained for the publication of this report accompanying images.

The Regional Ethics Review board at Uppsala University, Uppsala, Sweden approved the study (Approval number: 2009/414).

## Results

### Characteristics of staff in the two EDs

The gender distribution was evenly distributed in the groups at baseline, but there was a difference for the physicians at follow-up, as the proportion of men was higher at the county hospital, and the proportion of women was higher at the university hospital. The most frequent age group was 25–44 years, both at baseline and at follow-up, with a predominance of respondents aged 25–34 years except for the registered nurses/assistant nurses at the county hospital.

In all groups, the majority of staff had 1–5 years of experience in health care, except for the registered nurses/assistant nurses at the county hospital, who mostly had more than 21 years’ experience. The number of years of experience was more evenly distributed for the registered nurses/assistant nurses at the university hospital. The predominant duration of employment in the ED was 1–5 years, except for the physicians at the university hospital, where a greater proportion at follow-up had less than one years’ duration of employment (Table 2).

**Table 2:** Characteristics of the participating staff at the EDs at the two participating hospitals

	**County hospital**		**County hospital**		**University hospital**		**University hospital**	
	**Physicians**		**Registered nurses/assistant nurses**		**Physicians**		**Registered nurses/assistant nurses**	
	***n*** **= 86**	***n*** **= 92**	*n* **= 83**	***n*** **= 83**	***n*** **= 23**	***n*** **= 35**	***n*** **= 85**	***n*** **= 99**
	***n *** **(%)**		***n *** **(%)**		***n *** **(%)**		***n *** **(%)**	
	**Before**	**After**	**Before**	**After**	**Before**	**After**	**Before**	**After**
**Men**	44 (51)	59 (62)	17 (20)	15 (18)	11 (44)	14 (38)	18 (19)	23 (20)
**Women**	42 (49)	33 (34)	66 (79)	68 (81)	12(48)	21 (57)	67 (72)	76 (68)
**Age groups**								
18-24	-	-	4 (5)	1 (1)	-	-	4 (4)	2 (2)
25-34	41 (48)	36 (38)	20 (24)	22 (26)	12(48)	21 (57)	37 (40)	43 (38)
35-44	29 (34)	30 (31)	26 (31)	21 (25)	8 (32)	11 (30)	15 (16)	28 (25)
45-54	11 (13)	19 (20)	20 (24)	22 (26)	2 (8)	3 (8)	16 (17)	18 (16)
55-64	5 (6)	10 (10)	13 (16)	17 (20)	3 (12)	1 (3)	17 (18)	8 (7)
≥ 65	-	-	1 (1)	2 (2)	-	-	1 (1)	1 (1)
**Number of years in health care**								
< 1	6 (7)	2 (2)	1 (1)	-	2 (8)	2 (5)	5 (5)	4 (4)
1-5	34 (40)	33 (34)	20 (24)	11 (13)	10 40)	17 (46)	25 (27)	25 (22)
6-10	17 (20)	14 (15)	18 (21)	22 (26)	6 (24)	10 (27)	17 (18)	28 (25)
11-15	13 (15)	19 (20)	9 (11)	9 (11)	2 (6)	4 (11)	10 (11)	18 (16)
16-20	3 (4)	6 (6)	9 (11)	8 (10)	1 (4)	2 (5)	9 (10)	4 (4)
≥21	13 (15)	22 (23)	27 (32)	34 (41)	3 (12)	2 (5)	23 (25)	23 (20)
**Number of years you have worked in this unit**								
< 1	26 (30)	21 (22)	14 (17)	10 (12)	6 (24)	15 (41)	21 (23)	19 (17)
1-5	33 (38)	34 (35)	32 (38)	35 (42)	11(44)	13 (35)	26 (28)	43 (38)
6-10	14 (16)	16 (17)	16 (19)	13 (16)	4 (16)	8 (22)	24 (26)	20 (18)
11-15	3 (3)	13 (16)	6 (7)	11 (13)	1 (4)	1 (3)	4 (4)	12 (10)
16-20	4 (5)	4 (4)	7 (8)	4 (5)	-	-	4 (4)	2 (2)
≥21	3 (3)	7 (7)	8 (10)	11 (13)	-	-	7 (7.5)	4 (4)

### Changes in the dimensions between baseline and follow-up within each hospital

At the county hospital, there was a difference between baseline and follow-up in three of 14 dimensions. Two of these dimensions, *Team-work within hospital* and *Communication openness*, showed a positive change, whereas the score in *Information and support to staff at adverse events* was lower at follow-up.

At the university hospital, a difference was observed for five dimensions. Whereas two of the dimensions, *Team-work across hospital units* and *Team-work within hospital* showed a higher score, three dimensions were scored lower at follow-up, *Staffing, Information and support to patients at adverse events*, and *Patient safety grade*. Thus, the dimension *Team-work within hospital* was scored more positively at both EDs after the intervention (Table 3).

**Table 3:** Changes in the dimensions between baseline and follow-up within each hospital

**Dimension**	**County hospital**				**University hospital**			
	**Baseline**	**Follow-up**	**Change**	***p*-value**	**Baseline**	**Follow-up**	**Change**	***p*-value**
	**%**	**%**	**а**		**%**	**%**	**а**	
**All participants’ responses**	*n* 172	*n* 181			*n* 118	*n* 149		
1. Non-punitive response to error	31.8	31.9		NS	48.3	43.0		NS
2. Staffing	26.8	24.6		NS	52.1	45.9	-	*
3. Frequency of event reporting	21.9	23.6		NS	27.3	16.1		NS
4. Hospital management Support for patient safety	13.7	16.1		NS	36.9	34.7		NS
5. Team-work across hospital units	31.3	27.8		NS	34.7	43.0	+	***
6. Hospital Hand-off and transition	34.6	32.9		NS	46.8	46.6		NS
7. Information and support to patients at adverse events	44.8	40.1		NS	46.3	32.7	-	**
8. Information and support to staff at adverse events	37.6	29.7	-	*	28.2	31.7		NS
9. Overall perception of safety	24.3	27.1		NS	44.6	41.6		NS
11. Organizational learning-continuous improvement	37.4	35.9		NS	48.4	52.8		NS
12. Team-work within hospital	56.9	63.3	+	*	71.7	80.1	+	**
13. Communication openness	51.2	57.9	+	*	66.0	61.8		NS
14. Feedback and communication about error	45.8	50.1		NS	48.9	46.0		NS
15. Patient safety grade	62.0	56.7		NS	91.1	82.0	-	*

а Direction of the change from baseline measurement to follow-up.

*** = *p* < 0.001, ** = *p* < 0.01, * = *p* < 0.05, NS = not significant.

An index of < 50 is considered low and should lead to action, 51 - 69 suggests potential for improvement, and ≥ 70 indicates that the unit is functioning well.

### Changes in the dimensions within each hospital by each occupation

The physician group at the county hospital scored higher in three of 14 dimensions at the follow-up, *Overall perception of safety, Team-work within hospital*, and *Organizational learning–continuous improvement.*

For the physician group at the university hospital, a difference between baseline and follow-up was observed in two of the dimensions: *Team-work across hospital units* scored higher at follow-up, whereas *Staffing* scored lower at follow-up.

We observed a change in six of 15 dimensions for the registered nurses/assistant nurses at the county hospital. Two of these dimensions, *Safety culture dimension at unit level* and *Communication openness* scored higher, whereas the scores in *Team-work across hospital units*, *Information and support to patients at adverse events, Information and support to staff at adverse events*, and *Organizational learning–continuous improvement* scored lower at follow-up.

We observed a change in four of 15 dimensions for the registered nurses/assistant nurses at the university hospital. One of these dimensions, *Team-work within hospital*, showed a higher score at follow-up and three dimensions, *Frequency of event reporting*, *Information and support to patients at adverse events*, and *Patient safety grade* scored lower. The score for team-work increased for physicians at both hospitals and for registered nurses/assistant nurses at the university hospital (Table 4).

**Table 4:** Changes in the dimensions within each hospital by occupation

**Dimension**	**County hospital**				**University hospital**			
	**Base-line**	**Follow-up**	**Change**	**P-value**	**Base-line**	**Follow-up**	**Change**	**P-value**
**Target group**	***N 129***	***N 149***			***N 55***	***N 54***		
**Physicians’ responses**	*n* 86	*n* 96			*n* 25	*n* 37		
	%	%	а		%	%	а	
1. Non-punitive response to error	33.3	36.0		NS	49.7	43.2		NS
2. Staffing	28.3	28.1		NS	62.9	48.6	-	*
3. Frequency of event reporting	19.9	22.8		NS	23.0	15.0		NS
4. Hospital management support for patient safety	15.8	20.2		NS	42.0	41.8		NS
5. Team-work across hospital units	29.9	32.5		NS	29.8	42.8	+	*
6. Hospital hand-off and transition	29.8	33.3		NS	44.9	42.6		NS
7. Information and support to patients at adverse events	43.7	46.2		NS	40.2	33.3		NS
8. Information and support to staff at adverse events	32.4	29.3		NS	32.5	32.2		NS
9. Overall perception of safety	25.1	32.5	+	*	54.2	49.3		NS
11. Organizational learning-continuous improvement	28.0	37.3	+	*	55.3	61.1		NS
12. Team work within hospital	50.3	65.3	+	***	79.6	77.6		NS
13. Communication openness	55.3	56.8		NS	76.4	66.4		NS
14. Feedback and communication about error	34.8	42.4		NS	54.0	46.8		NS
15. Patient safety grade	73.3	66.3		NS	91.7	88.9		NS
**Target group**	***N 108***	***N 114***			***N 125***	***N 125***		
**Registered nurses’ + assistant nurses’ responses**	***n *** **86**	***n *** **85**			***n *** **93**	***n *** **112**		
	**%**	**%**			**%**	**%**		
1. Non-punitive response to error	30.3	27.4		NS	47.9	42.9		NS
2. Staffing	25.2	20.8		NS	49.2	44.9		NS
3. Frequency of event reporting	24.0	24.5		NS	28.6	16.4	-	**
4. Hospital management support for patient safety	11.6	11.6		NS	35.3	32.1		NS
5. Team work across hospital units	32.8	22.6	-	**	36.1	43.0		NS
6. Hospital hand-off and transition	39.4	32.5		NS	47.4	48.1		NS
7. Information and support to patients at adverse events	45.8	34.2	-	**	48.3	32.6	-	***
8. Information and support to staff at adverse events	42.6	30.1	-	*	26.7	31.5		NS
9. Overall perception of safety	23.4	21.3		NS	42.0	39.0		NS
10. Safety culture dimension unit level	40.5	52.3	+	**	56.5	52.7		NS
11. Organizational learning-continuous improvement	46.1	34.5	-	**	46.6	50.0		NS
12. Team- work within hospital	63.7	61.0		NS	69.6	81.0	+	***
13. Communication openness	47.1	59.1	+	**	63.0	60.2		NS
14. Feedback and communication about error	56.7	58.3		NS	47.5	45.7		NS
15. Patient safety grade	50.1	46.2		NS	90.9	79.6	-	*

а Direction of the change from baseline measurement to follow-up . *** = *p* < 0.001, ** = *p* < 0.01, * = *p* < 0.05, NS = not significant. An index of < 50 is considered low and should lead to action, 51 - 69 suggests potential for improvement, and ≥ 70 indicates that the unit is functioning well.

### Changes in the dimensions between occupations at baseline and follow-up by hospital

At the county hospital, a difference between physicians and nurses/assistant nurses was observed at baseline in five of 14 dimensions. The nurses/assistant nurses had a higher score in four dimensions, *Hospital hand-off and transition, Organizational learning–continuous improvement, Team-work within hospital*, and *Feedback and communication about error*, and they had a lower score in *Patient safety grade*.

At the follow-up, eight dimensions differed between the physicians and the nurses/assistant nurses. The physicians scored higher in seven dimensions*, Non-punitive response to error*, *Staffing, Hospital management support for patient safety, Team-work across hospital units, Hospital hand-off and transition, Information and support to patients at adverse events, Overall perception of safety*, and *Patient safety grade*. They scored lower in *Feedback and communication about error*, which was the only dimension for which physicians scored lower than nurses/assistant nurses at both baseline and follow-up. At the university hospital, a difference between physicians and nurses/assistant nurses was observed at baseline in three of 14 dimensions. The physicians scored higher in *Staffing, Overall perception of safety*, and *Communication openness*. At follow-up, the physicians scored higher in two dimensions, *Overall perception of safety and organisational learning-continous improvement. Overall perception of safety* was the only dimension for which physicians scored higher than nurses/assistant/nurses at both baseline and follow-up (Table 5).

**Table 5:** Changes in the dimensions between occupations at baseline and follow-up by hospital

**Dimension**	**Baseline**			**Follow up**		
	**Physicians**	**Registered nurses/assistant nurses**	**P-value**	**Physicians**	**Registered nurses/assistant nurse**	**P-value**
**Target group**	***N 129***	***N 108***		***N 149***	***N 114***	
**County Hospital responses**	***n *** **86**	***n *** **86**		***n *** **96**	***n *** **85**	
	%	%		%	%	
1. Non-punitive response to error	33.3	30.3	NS	36.0	27.4	*
2. Staffing	28.3	25.2	NS	28.1	20.8	*
3. Frequency of event reporting	19.9	24.0	NS	22.8	24.5	NS
4. Hospital management support for patient safety	15.8	11.6	NS	20.2	11.6	**
5. Team-work across hospital units	29.9	32.8	NS	32.5	22.6	**
6. Hospital hand-off and transition	29.8	39.4	**	33.3	32.5	NS
7. Information and support to patients at adverse events	43.7	45.8	NS	46.2	34.2	**
8. Information and support to staff at adverse events	32.4	42.6	NS	29.3	30.1	NS
9. Overall perception of safety	25.1	23.4	NS	32.5	21.3	***
11. Organizational learning-continuous improvement	28.0	46.1	***	37.3	34.5	NS
12. Teamwork within hospital	50.3	63.7	***	65.3	61.0	NS
13. Communication openness	55.3	47.1	NS	56.8	59.1	NS
14. Feedback and communication about error	34.8	56.7	***	42.4	58.3	**
15. Patient safety grade	73.3	50.1	**	66.3	46.2	**
**Target group**	***N 55***	***N 125***		***N 54***	***N 125***	
**University Hospital responses**	***n *** **25**	***n *** **93**		***n *** **37**	***n *** **112**	
	***%***	***%***		***%***	***%***	
1. Non-punitive response to error	49.7	47.9	NS	43.2	42.9	NS
2. Staffing	62.9	49.2	**	48.6	44.9	NS
3. Frequency of event reporting	23.0	28.6	NS	15.0	16.4	NS
4. Hospital management support for patient safety	42.0	35.3	NS	41.8	32.1	NS
5. Team-work across hospital units	29.8	36.1	NS	42.8	43.0	NS
6. Hospital hand-off and transition	44.9	47.4	NS	42.6	48.1	NS
7. Information and support to patients at adverse events	40.2	48.3	NS	33.3	32.6	NS
8. Information and support to staff at adverse events	32.5	26.7	NS	32.2	31.5	NS
9. Overall perception of safety	54.2	42.0	**	49.3	39.0	*
11. Organizational learning-continuous Improvement	55.3	46.6	NS	61.1	50.0	*
12. Teamwork within hospital	79.6	69.6	NS	77.6	81.0	NS
13. Communication openness	76.4	63.0	**	66.4	60.2	NS
14. Feedback and communication about error	54.0	47.5	NS	46.8	45.7	NS
15. Patient safety grade	91.7	90.9	NS	88.9	79.6	NS

*** = *p* < 0.001, ** = *p* < 0.01, * = *p* < 0.05, NS = not significant. An index of < 50 is considered low and should lead to action, 51 - 69 suggests potential for improvement, and ≥ 70 indicates that the unit is functioning well.

## Discussion

The main findings were that the staff at both hospitals scored more positively in the dimension *Team-work within hospital* after implementing a new working model, aiming to improve patient flow in the ED. An improvement was also seen in the dimension *Communication openness* at the county hospital and *Team-work across units at the university hospital.*

For the two overall questions *Patient safety grade* and *Overall perception of patient safety* there was a tendency for both physicians and registered nurses/assistant nurses to estimate a lower value after the quality improvement, except for the physicians at the county hospital who estimated a significantly higher value for *Overall perception of safety*.

### Quality improvement with different implementation strategies

Improvement for the dimensions *Team-work across hospital* and *Team-work within hospital* can likely be explained by the quality improvement project that was based on lean principles, of which one is “building in quality”, which emphasizes team-work. The role for each team member, as well as the organization of patient flow, is one of the major focuses of improvements using lean principles [29]. The relatively small improvements in performance between the first and second measurements in the present study may be because no special efforts were made in the form of leadership training or team training, both of which are well-documented measures for patient safety improvement [6]. The quality improvement project resulted in no significant difference in the dimensions concerning *Overall patient safety*. However, the university hospital results showed a lower score for the dimension *Patient safety grade* at follow-up. These poor results for *Patient safety grade* may be explained by the top-down initiation of the change process, and hence the lack of support at the unit level. As the main purpose of the project was to improve patient flow and the working environment, the aspect of patient safety may not have been communicated very clearly to the staff.

Another explanation for the observed results may be that the quality improvement project was not fully implemented at the county hospital at the time of follow-up, and that only one year had elapsed between the quality improvement project and the follow-up. Implementation studies show that the effect of quality improvement is relatively low during the first year after implementation [32].

There were different implementation strategies at the two EDs, which may explain the different results at the two hospitals. At the county hospital, the quality improvement project was initiated by the physicians from a bottom-up perspective, whereas at the university hospital, the initiative was top-down with external facilitators, giving little room for the staff to influence quality improvement. As health care is regarded as a complex system, it is important to use comprehensive approaches targeting different levels, settings and groups to enhance the implementation effect [13,32].

One factor that may have complicated the implementation process could be the steady increase in patient numbers at both EDs during recent years. It was shown in a previous study that staff frustration was accentuated when the waiting time at the ED is considered non-acceptable [33]. In the same study it was shown that much of this frustration also were connected to issues concerning leadership, work organization, and lack in patient safety culture, which also could have been a complicating factor for the implementation.

Another finding was that the staff at the university hospital scored higher than the staff at the county hospital in all dimensions at both measurements except for the dimensions *Frequency of event reporting, Information and support to patients after adverse events*, and *Feedback and communication about error*. These findings demonstrate the important role played by the hospital management in achieving patient safety. Frequent contributors to medical injuries in health care are failures in communication and team-work [4]. Improved team-work is beneficial for communication, and vice versa [5]. This may explain the positive result for the dimension *Communication openness* at the county hospital.

### The effect of quality improvement by occupation

#### Physicians

The improved results for the physicians at the county hospital in the dimensions *Overall perceptions of safety, Team-work within unit*, and *Organizational learning–continuous improvement* may suggest that they had better control and supervision of the patients and the patient flow. Currently, senior physicians working in a team are in the first receiving line for patients, instead of the previous regimen with initial nursing triage and junior physicians at the second stage. The co-operation between staff groups has also increased. Moreover, as previously addressed, the quality improvement process at this hospital was based on initiatives by internal medicine physicians.

Physicians at the university hospital improved their scores in the dimension *Team-work across hospital*. One explanation for this may be the new model of working in accordance with lean principles, which, as previously mentioned, emphasize team-work. In this case, it may also have affected team-work across the hospital, as the physicians in the ED need to co-operate with both specialists from other departments within the hospital and with nurses in different units in the hospital to enhance a better flow between the ED and these units. Another factor that may have influenced the results is that the physicians are responsible for hand-off communication. The negative change at the university hospital in the dimension *Staffing* may be interpreted as an expression of increased work-load, as well as difficulties in having a specialist in place in every team for all shifts. An additional explanation may be the difference in experience of the staff at the unit between baseline and follow-up, as at follow-up there were an increased proportion of physicians who had worked in the ED for less than one year.

#### Registered nurses/assistant nurses

The positive change in the dimensions *Safety culture dimension unit level* and *Communication openness* may be because of the new organization structure at the county hospital, with senior physicians in the initial receiving line for patients and an emphasis on team-work instead of the previous model. At the university hospital, registered nurses/assistant nurses scored *Team work within hospital* higher at the follow-up, which was in accordance with the physicians at the same ED.

Negative changes were shown for *Team-work across hospital units, Information and support to staff at adverse events*, and *Organizational learning–continuous improvement* at the county hospital and for *Frequency of event reporting* and *Patient safety grade* at the university hospital. In addition, registered nurses/assistant nurses at both hospitals scored lower at follow-up for *Information and support to patients at adverse events*. One explanation for some of the negative results may be that the quality improvement process did not include any team-training program or communication programs, which were linked with positive results in two other studies [4,6]. One of these reported a positive effect on two of 12 dimensions of HSOPSC, *Frequency of event reporting* and *Organizational learning*, after a quality improvement project including communication team training, using Team-STEPPS [4]. The other study showed positive changes in four dimensions of HSOPSC, *Team-work within unit, Feedback and communication, Communication openness*, and *Overall patient safety grade* after quality improvement with the same Team-STEPPS training program [6].

### Differences between occupations at baseline and at follow-up

#### County hospital

At baseline, the results for the county hospital showed a higher score in four of five dimensions for registered nurses/assistant nurses compared with physicians. At the follow-up, the physicians scored higher than registered nurses/assistant nurses in eight dimensions. One of these dimensions was *Overall perception of safety*. The higher score could be explained by the introduction of specialists instead of junior physicians as the initial recipients of the patients. The same explanation may be valid for the scores for *Patient safety grade*. Although both groups scored lower at follow-up, the physicians still scored higher than registered nurses/assistant nurses.

#### University hospital

At the university hospital, the physicians are employed at the ED, which may explain why there were few differences between registered nurses/assistant nurses and physicians both at baseline and follow-up. Thus, both groups shared the staffing conditions, and they all shared the same daily environment and the same values set by the department. One of the dimensions, *Patient safety grade* was scored higher by the physicians both at baseline and follow-up, which could be an expression of the physicians’ role in the team, as, in contrast to the registered nurses/assistant nurses, they are in control of the treatment plan for the patients and hence able to plan the next move. It is also possible that the treatment plan may not been sufficiently communicated to the team.

### Limitations

Measuring patient safety culture by a questionnaire is self-limiting, as patient safety culture is a multifaceted concept. It has been shown that it is difficult to define measurable components of patient safety culture [20], illustrated by the fact that a number of different patient safety climate questionnaires have been developed [21,22]. One way to overcome this is to use a mixed method design, which was not used in this study.

Another limiting factor of this study was that the study groups were not identical at the two measurement time points. Another factor that may have influenced the result was that a higher proportion of physicians at the university hospital than at the county hospital had worked in the ED for less than one year. Only a few positive differences were seen between baseline and follow-up, which may be explained by a limited effect of the quality improvement project itself. It is possible that the result may have been improved in further dimensions for all the staff if the quality improvement project had included areas such as communication skills and team-work training together with the changes to improve patient flow, and provided that the implementation process had been systematic. The time span between the quality improvements and the second measurement could also be questioned, since changes take time.

## Conclusion

The result showed improvements relating to team-work and communication openness. Most of the improvements at follow-up were seen for the physicians, mainly at the county hospital in dimensions *Overall perception of safety, Team-work within hospital*, and *Organisational learning-continuous improvement*. Overall, the physicians at the university hospital scored higher in the majority of the dimensions than those at the county hospital. The low number of positive changes seen could have been influenced by the lack of team training and communication programs in connection with the implementation of the new work model, which previous studies have shown to be of importance. Further, a cultural change is challenging and takes time, and the time from implementation to follow-up may have been too short for the staff to experience any effect on patient safety.

## Competing interests

None of the authors report any competing interests.

## Authors’ contributions

LB, AL, M-LE and ME conceived the study, collected data, analysed and interpreted data and drafted the manuscript. AB analysed and interpreted data and drafted the manuscript. All authors read and approved the final manuscript.

## Pre-publication history

The pre-publication history for this paper can be accessed here:

http://www.biomedcentral.com/1472-6963/14/296/prepub
